# Protein Arginine Methyltransferase 5 Functions in Opposite Ways in the Cytoplasm and Nucleus of Prostate Cancer Cells

**DOI:** 10.1371/journal.pone.0044033

**Published:** 2012-08-27

**Authors:** Zhongping Gu, Yirong Li, Peng Lee, Tao Liu, Chidan Wan, Zhengxin Wang

**Affiliations:** 1 Department of Cancer Biology, The University of Texas MD Anderson Cancer Center, Houston, Texas, United States of America; 2 Department of Thoracic Surgery, Tangdu Hospital, Fourth Military Medical University, Xi'an, China; 3 Department of Pathology and Urology, Langone Medical Center, New York University, New York, United States of America; 4 Department of General Surgery, Union Hospital, Tongji Medical College, Huazhong University of Science and Technology, Wuhan, Hubei, P. R. China; 5 Center of Pancreatic Disease , Department of General Surgery, Union Hospital, Tongji Medical College, Huazhong University of Science and Technology, Wuhan, Hubei, P. R. China; University of Colorado, United States of America

## Abstract

Protein arginine methyltransferase 5 (PRMT5) plays multiple roles in a large number of cellular processes, and its subcellular localization is dynamically regulated during mouse development and cellular differentiation. However, little is known of the functional differences between PRMT5 in the cytoplasm and PRMT5 in the nucleus. Here, we demonstrated that PRMT5 predominantly localized in the cytoplasm of prostate cancer cells. Subcellular localization assays designed to span the entire open-reading frame of the PRMT5 protein revealed the presence of three nuclear exclusion signals (NESs) in the PRMT5 protein. PRMT5 and p44/MED50/WD45/WDR77 co-localize in the cytoplasm, and both are required for the growth of prostate cancer cells in an PRMT5 methyltransferase activity-dependent manner. In contrast, PRMT5 in the nucleus inhibited cell growth in a methyltransferase activity-independent manner. Consistent with these observations, PRMT5 localized in the nucleus in benign prostate epithelium, whereas it localized in the cytoplasm in prostate premalignant and cancer tissues. We further found that PRMT5 alone methylated both histone H4 and SmD3 proteins but PRMT5 complexed with p44 and pICln methylated SmD3 but not histone H4. These results imply a novel mechanism by which PRMT5 controls cell growth and contributes to prostate tumorigenesis.

## Introduction

Protein arginine methyltransferase 5 (PRMT5) is a type II protein arginine methyltransferase that catalyzes the symmetrical dimethylation of arginine residues within target proteins [1]. PRMT5 is highly conserved among yeast, animals, and higher plants and has been implicated in diverse cellular and biological processes, including transcriptional regulation [2], [3], [4], RNA metabolism [1], [5], ribosome biogenesis 6], Golgi apparatus structure maintenance [7], and cell cycle progression [2]. PRMT5 is also involved in germ cell formation, specification, and maintenance [8], [9], [10], [11], [12], [13]. In mammalian cells, PRMT5 localizes to both the cytoplasm and the nucleus, and it methylates multiple histone and nonhistone proteins [1]. In the nucleus, PRMT5 has been found in the SWI/SNF and NURD chromatin-remodeling complexes [14], [15], where it methylates histones as well as transcription factors/regulators [2], [3], [4]. In the cytoplasm, PRMT5 forms a 20S protein arginine methyltransferase complex, termed the “methylosome,” consisting of spliceosomal snRNP Sm proteins, PRMT5, pICln, and WD repeat protein (MEP50/WD45) [16], [17], [18]. In this complex, PRMT5 methylated Sm proteins [16], [19], and such methylation increased the binding affinity of these Sm proteins for the survival motor neuron (SMN), the spinal muscular atrophy disease gene product [20], [21]. Subsequently, the PRMT5- and SMN-complexes cooperate to load the Sm proteins onto U snRNAs, forming U snRNPs [22]. Although *in vitro* biochemical evidence indicated that symmetric arginine dimethylation is essential for pre-mRNA splicing [23], to what extent PRMT5 affects splicing *in vivo* remains elusive. PRMT5 is crucial for mouse embryonic development [8].

We purified and cloned a novel androgen receptor (AR)-interacting protein, designated p44 [24], [25]. The protein sequence of p44 is identical to that of a component (MEP50) of the methylosome complex [18] and a subunit (WD45) of the SMN complex [17]. The p44 protein contains 342 amino acid residues and seven putative WD-40 repeats and is also designated WDR77 in the gene bank (Accession:AAH9411.1). It interacts with AR and regulates expression of a set of androgen target genes in the prostate gland and in prostate cancer [24], [25], [26], [27]. The p44 protein localizes in the cytoplasm of prostate epithelial cells of mice younger than 28 days; p44 nuclear translocation begins at age 28 days and is completed at age 45 days [28]. Nuclear translocation of p44 is correlated with a dramatic decrease in the proliferation rate of epithelial cells [28] and with functional cytodifferentiation of luminal cells, occurring with the expression of the prostate-specific secretory proteins [29], [30], [31], [32]. Thus, p44 cytoplasmic localization is associated with prostate epithelial cell proliferation, whereas its nuclear localization is associated with epithelial cell differentiation. Immunohistochemical staining of prostate specimens showed that the p44 protein localizes in the nucleus of benign epithelial cells and in the cytoplasm of prostate cancer cells [25]. Translocation of p44 from the nucleus to the cytoplasm occurs in prostatic intraepithelial neoplasia and prostate cancer lesions [25], [26]. Forced nuclear localization of p44 inhibited growth of prostate cancer cells in tissue culture [25] and completely abolished the growth of prostate tumor xenografts in nude mice [26]. This growth inhibition was associated with upregulation of *p21* and *p27* gene expression; downregulation of *cyclin A*, *cyclin B*, and *CDK2* gene expression; and cell cycle arrest at the G_1_/G_0_ phase [25], [26]. Thus, p44 function is regulated by its subcellular localization.

**Figure 1:**
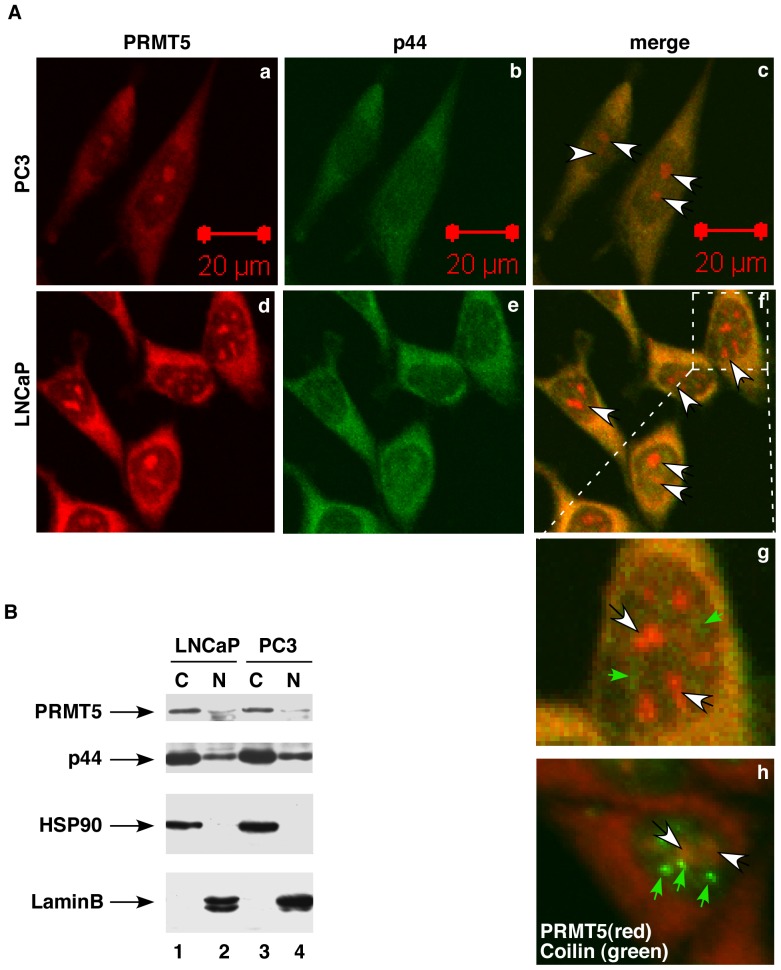
PRMT5 localized with p44 in the cytoplasm but not in the nucleus in prostate cancer cells. (**A**) PC3 and LNCaP cells were immunohistochemically stained with anti-PRMT5 and -p44 antibodies (panels a-g) or anti-PRMT5 plus -coilin antibodies (panel h). The fluorescent signals were observed under a confocal microscope with a red filter (to detect PRMT5) or green filter (to detect p44 or coilin). Right panels show merged images of PRMT5 and p44 or coilin staining. White and green arrowheads indicate PRMT5 and p44 or coilin signals in the nucleus, respectively. (**B**) Western blot of cytoplasmic and nuclear fractions of LNCaP and PC3 cells with anti-PRMT5, -p44, -HSP90, or anti-lamin B antibody. C, cytoplasm; N, nucleus.

PRMT5 forms a stoichiometric complex with p44/MEP40/WD45/WDR77 in various cells [33], [34], [35], and its subcellular localization is dynamically regulated during mouse development [8]. The functional role of PRMT5 in the cytoplasm and nucleus and the relationship of its subcellular localization to prostate cancer have not been investigated. In the current study, we found that cytoplasmic PRMT5 is essential for the growth of prostate cancer cells, whereas nuclear PRMT5 inhibits prostate cancer cell growth. Consistent with these observations, PRMT5 localizes in the nucleus in benign prostate epithelial cells and, in contrast, localizes in the cytoplasm in premalignant and cancerous prostate tissues. Therefore, the PRMT5 function is regulated by its subcellular localization, and this nucleocytoplasmic transport may play an important role in prostate tumorigenesis.

**Figure 2:**
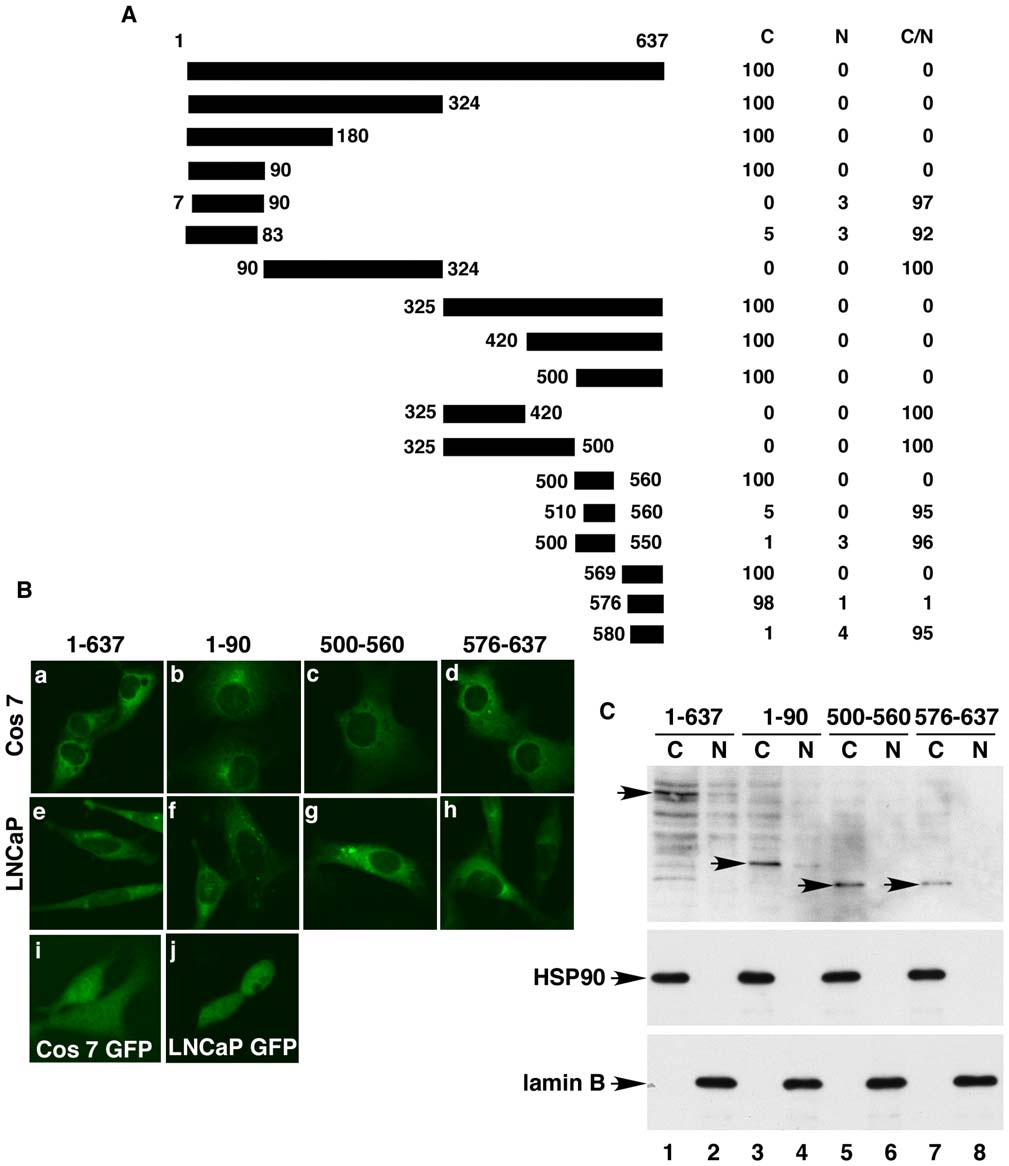
PRMT5 contains three nuclear exclusion signals. (**A**) Diagrams of the PRMT5 truncations expressed as GFP-fusion proteins. The percentages of cells with GFP-PRMT5 truncations in the cytoplasm (C), nucleus (N), or cytoplasm plus nucleus (C/N) are shown on the right. (**B**) Subcellular localization of isolated nuclear export signals. Cells were transfected with pcDNA-f:GFP-PRMT5, -PRMT5(1–90), -PRMT5(500–560), -PRMT5(576–637), or pcDNA-GFP and observed under a confocal microscope. (**C**) Western blot analysis of cytoplasmic and nuclear fractions of cells transfected with pcDNA-f:GFP-PRMT5, -PRMT5(1–90), PRMT5(500–560), or PRMT5(576–637) with anti-FLAG, -HSP90, or -lamin B antibody.

## Materials and Methods

Our research did not involve human participants and animals, only the use of human prostate cancer tissues. The patients cannot be identified, directly or indirectly, through identifiers linked to the subjects. Thus, the research project was exempt under Exemption 4 (45 CFR Part 46) and an ethics statement is not required.

**Figure 3:**
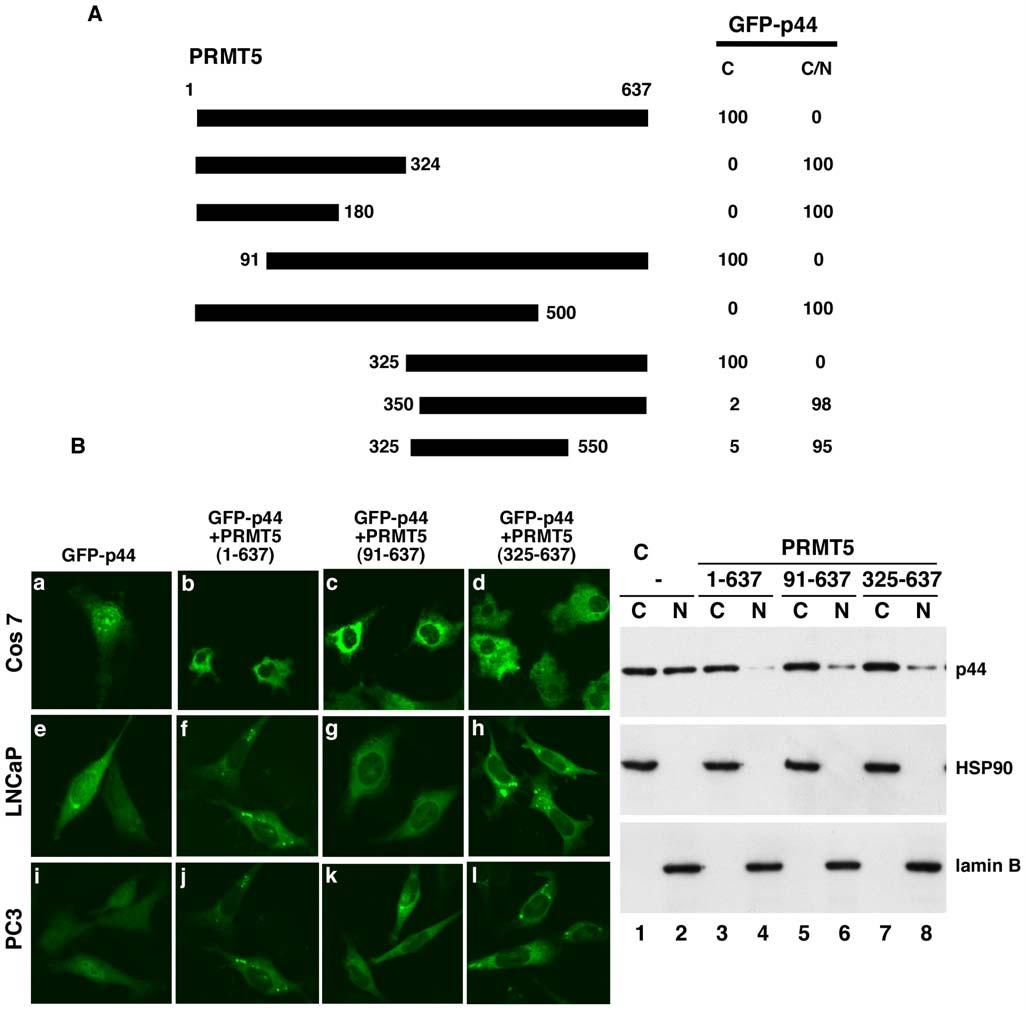
PRMT5 promotes p44 cytoplasmic translocation. (**A**) Diagrams of the PRMT5 truncations. Cells were transfected with pcDNA-GFP-p44 and pcDNA-PRMT5 or pcDNA-PRMT5 truncations, and the percentages of cells with GFP-p44 in cytoplasm (C) or cytoplasm plus nucleus (C/N) are shown on the right. (**B**) Cytoplasmic translocation of GFP-p44 driven by PRMT5. Cells were transfected with pcDNA-GFP-p44 alone or together with pcDNA-f:PRMT5, -f:PRMT5(91–637), or f:PRMT5(325–637), and the GFP-p44 subcellular localization was observed under a confocal microscope. (**C**) Western blot analysis of cytoplasmic and nuclear fractions of Cos 7 cells described in B with anti-p44, -HSP90, or -lamin B antibody.

### Prostate Cancer Samples and Immunohistochemistry

Benign and cancerous prostate tissues were derived from radical prostatectomy specimens of 19 patients with prostate cancer treated at New York University Medical Center, and the study protocol was approved by its institutional review board. Patient identities were removed from all samples and an exemption from the need for consent was granted by the institutional review board of New York University School of Medicine, so no informed consent was needed. The tissues were fixed in 10% neutral buffered formalin and embedded in paraffin. Immunohistochemical analysis was performed on the 19 human prostate cancer samples as described previously [36], [37]. Antibodies (anti-p44 antibody, 1∶50; anti-PRMT5 antibody, 1∶20; from BD Transduction Laboratories) were applied to the slide sections and incubated overnight. A streptavidin–biotin peroxidase detection system with 3,3′-diaminobenzidine as substrate was used according to the manufacturer's instructions (DAKO A/S, Grostrup, Denmark).

**Figure 4:**
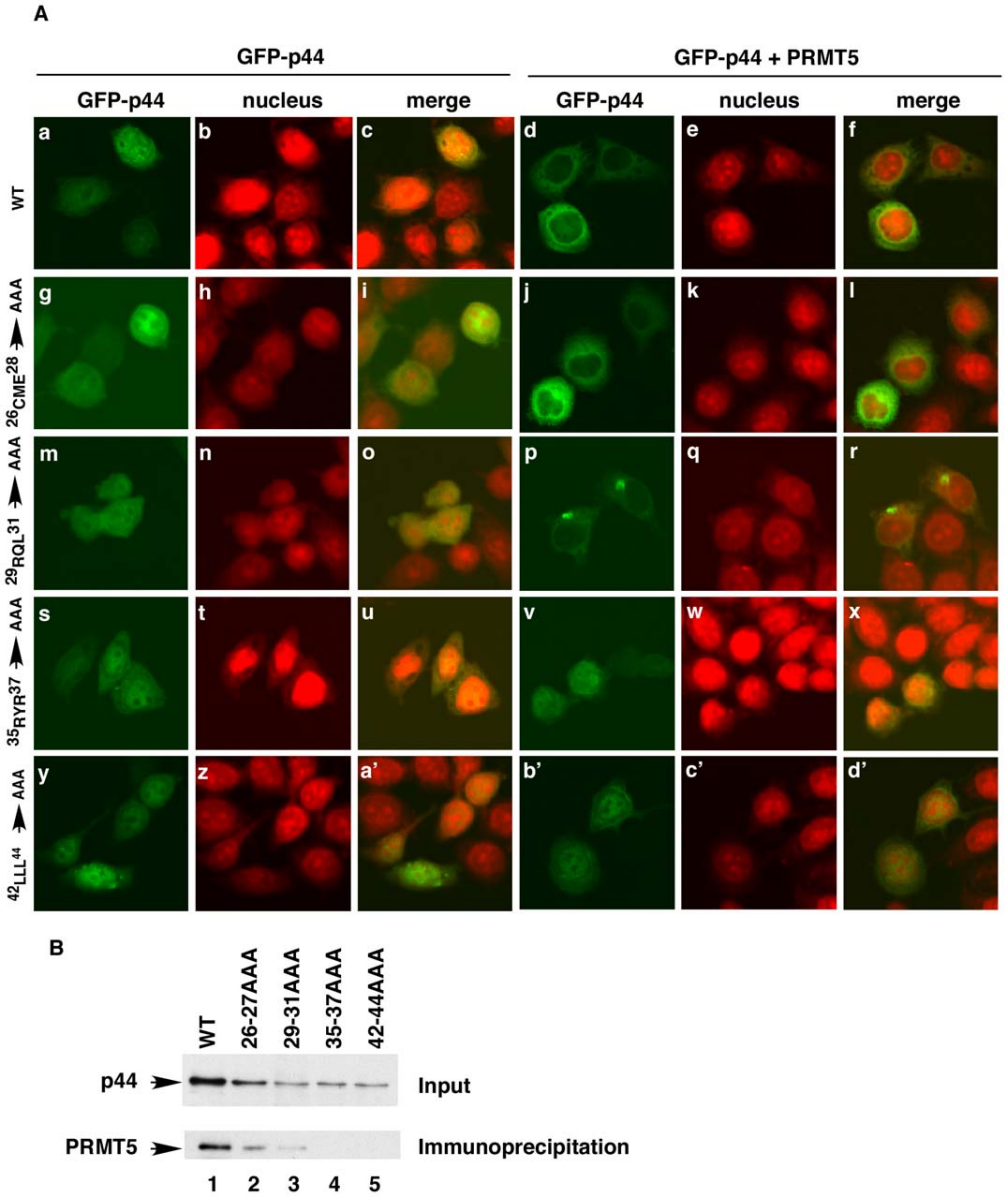
The PRMT5-p44 interaction is essential for the PRMT5-promoted p44 cytoplasmic localization. (**A**) Mutations in p44 abolished PRMT5-driven p44 cytoplasmic translocation. Cells were transfected with pcDNA-GFP-p44(WT) or pcDNA-GFP-p44(MT) alone or together with pcDNA-PRMT5. The nucleus was stained with Far-red, and the subcellular localization of GFP-p44 was observed under a confocal microscope. (**B**) Mutations in p44 abolished the interaction of p44 with PRMT5. Cells were transfected with pcDNA–f:p44 (WT) (lane 1) or pcDNA–f:p44 (MT) (lanes 2–5), and whole-cell lysates were prepared for immunoprecipitation with anti-FLAG antibody (M2 agarose). Western blot with anti-PRMT5 was performed to detect the precipitated PRMT5 (bottom panel). Top panel shows expression of wild-type (WT) or mutated (MT) p44 in the lysates used for the immunoprecipitation.

Cultured cells were grown on chamber slides and fixed with cold methanol (–20°C) for 10 min. Nonspecific proteins were blocked in 4% fish gelatin in PBS for 20 min. Overnight incubation at 4°C with primary antibodies was performed followed by a 1-h incubation with anti-mouse or anti-rabbit IgG antibody labeled with Alexa 595 (1∶500; Invitrogen) at room temperature. The samples were washed in PBS and then counterstained with TOPRO 3, Far-red, or Sytox green (Molecular Probes) for 10 min at room temperature, mounted in Histogel (Linaris Histogel), and analyzed directly by fluorescence confocal microscopy. For double staining, anti-PRMT5 and anti-p44 or anti-PRMT5 and anti-coilin (1∶100, ProteinTech) were incubated with cells overnight at 4°C. The secondary antibodies (anti-rabbit IgG labeled with DyLight 488, 1∶1,000, and anti-mouse IgG labeled with DyLight 649, 1;1,000) were used.

**Figure 5:**
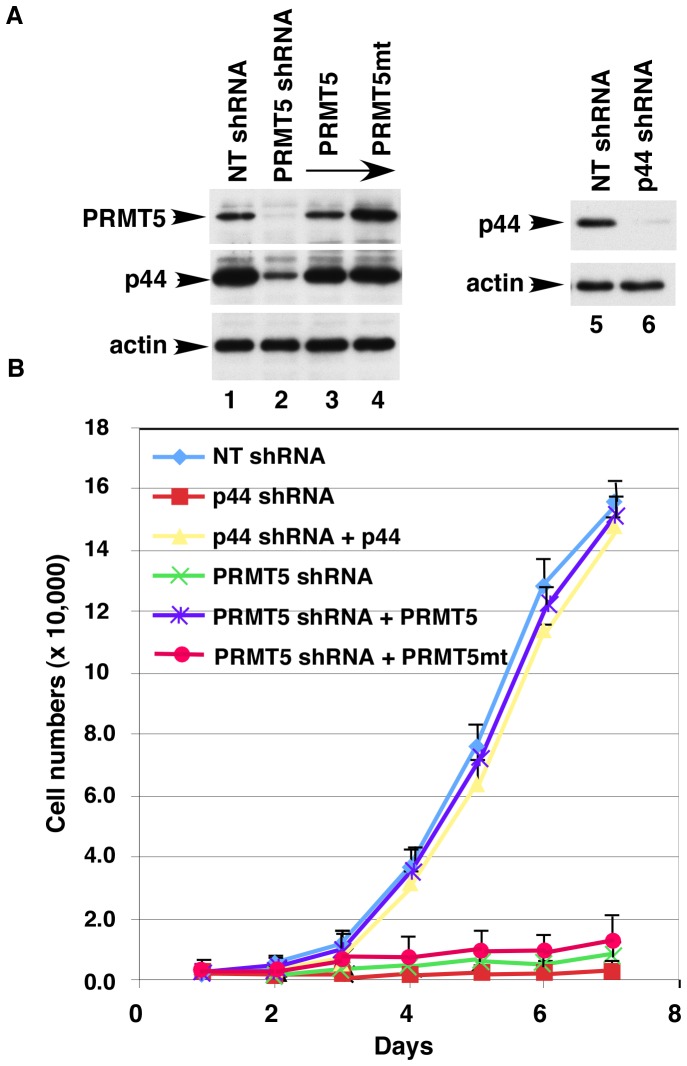
Silencing PRMT5 or p44 expression inhibited the growth of prostate cancer cells. (**A**) The shRNA-mediated silencing of PRMT5 or p44 expression in prostate cancer cells. Western blot analysis of whole-cell lysates made from LNCaP cells infected with lentivirus expressing the non-target (NT) shRNA (lanes 1, 5), PRMT5 (lanes 2–4), or p44 (lane 6) shRNAs. The shRNA-resistant PRMT5 (lane 3) or PRMT5 R368A mutant (lane 4) was expressed in the PRMT5-expressing LNCaP cells. (**B**) Growth curves of prostate cancer cells expressing NT shRNA, PRMT5 shRNAs, p44 shRNA, PRMT5 shRNAs plus PRMT5, p44 shRNA plus p44, or PRMT5 shRNAs plus PRMT5mt.

### Cell Culture and Growth Assay

LNCaP, PC3, and Cos 7 cells were cultured in RPMI 1640 medium (Cellgro) with 10% (v/v) fetal bovine serum (HyClone). For the cell growth assay, cells (5,000 per well) were plated onto 24-well plates, and cell numbers were counted every day for 7 days.

**Figure 6:**
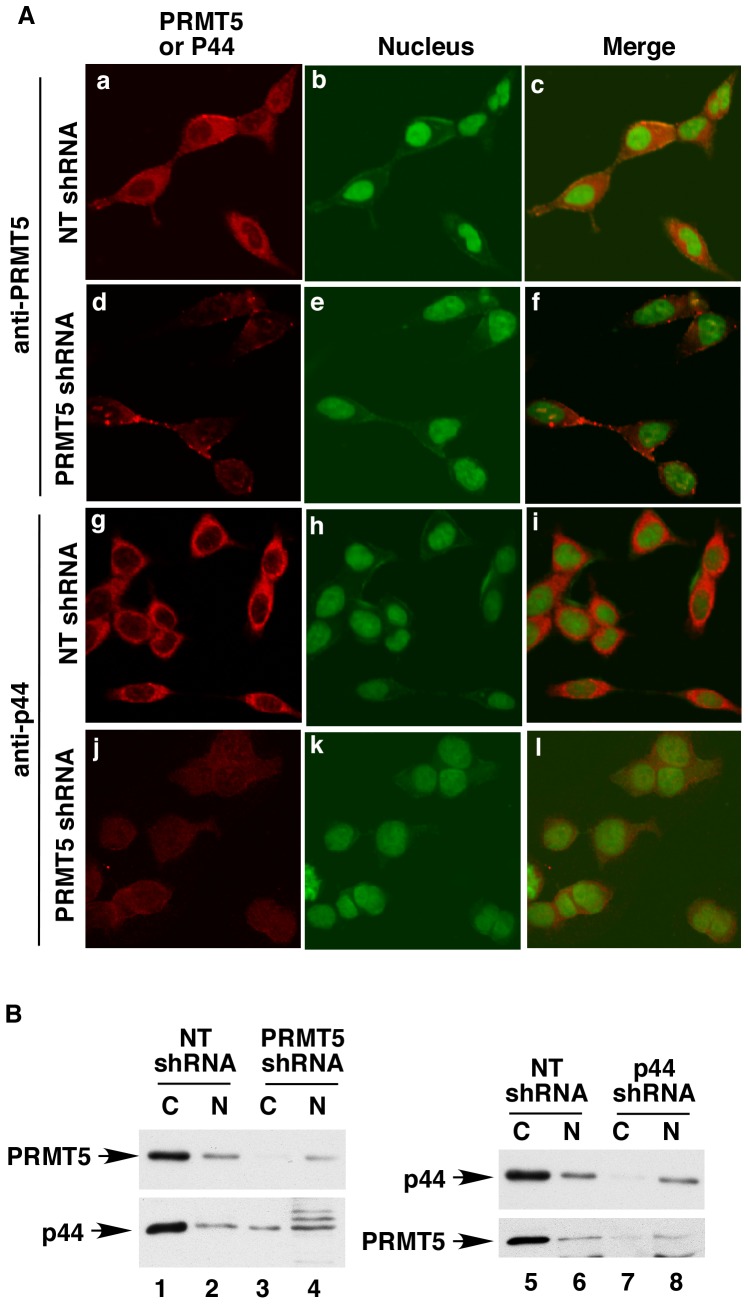
PRMT5 and p44 co-exist in the cytoplasm. (**A**) Silencing PRMT5 expression decreased p44 protein levels in the cytoplasm. LNCaP cells were infected with NT-shRNA or PRMT5 shRNA and immunostained with anti-PRMT5 or -p44 antibody. The nucleus was counterstained with Sytox green (middle panels, green). The samples were observed under a confocal microscope. (**B**) Western blot of cytoplasmic (C) and nuclear (N) fractions of LNCaP cells expressing NT-shRNA, PRMT5 shRNA, or p44 shRNA with anti-p44 or anti-PRMT5 antibody.

### DNA Constructs and Transient Transfection

The PRMT5 cDNA fragments were amplified from the pcDNA-PRMT5 construct [24] and subcloned into the pcDNA-f:GFP construct [28] to express the N-terminal f:GFP-fusion proteins of PRMT5 truncations. All constructs were verified by restriction enzyme digestion and by DNA sequencing. The strong nuclear localization signal (RKKKRKV) was fused at the N-terminal end of PRMT5 to express the NLS-PRMT5 fusion protein. DNA constructs (1 microgram for each construct) were transiently transfected into LNCaP, PC3, or Cos 7 cells (1×10^5^) using Lipofectamine 2000 (Invitrogen) following the manufacturer's instructions. The transfected cells were fixed with cold (–20°C) methanol for 10 min, stained with TO-PRO 3 (10 micrograms/ml) (Molecular Probes), mounted in Histogel (Linaris), and analyzed directly by fluorescence confocal microscopy.

**Figure 7:**
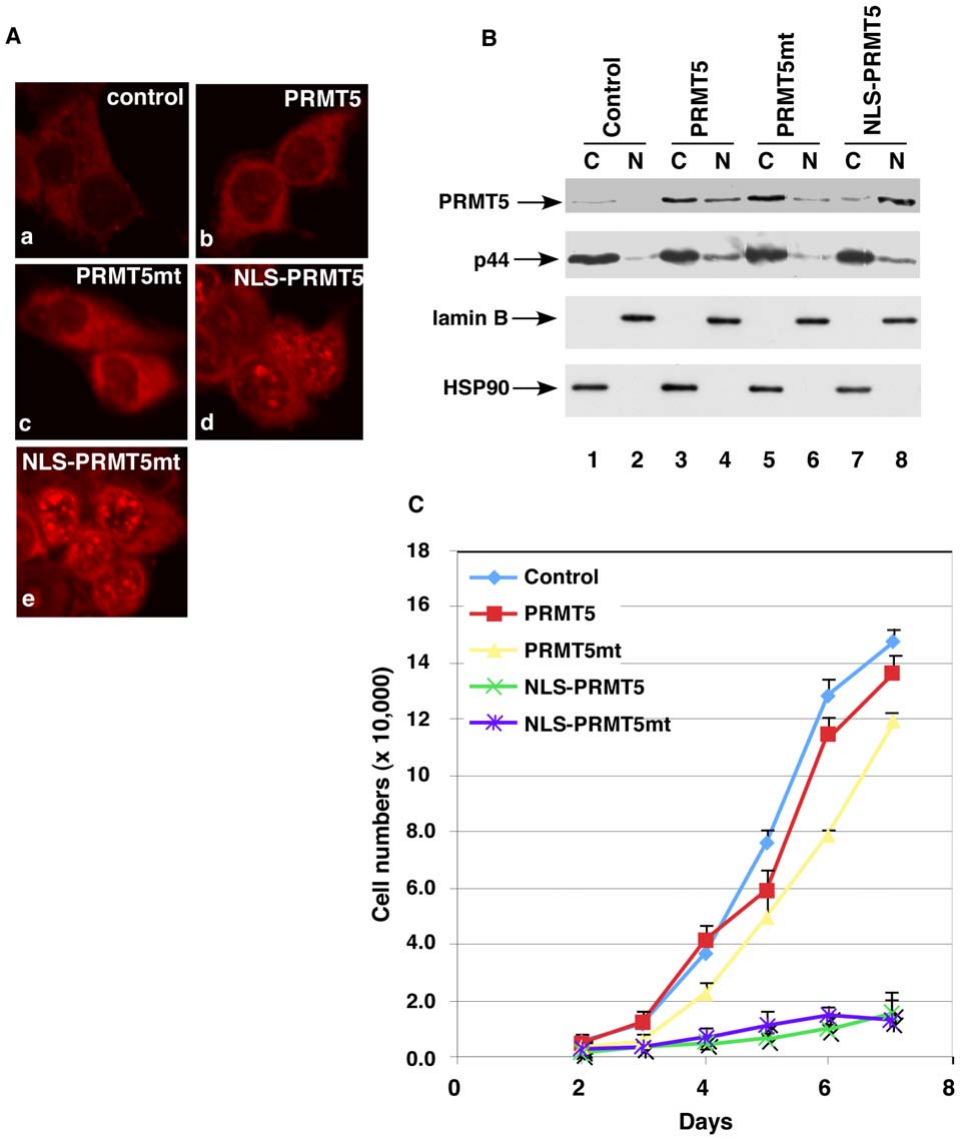
The nuclear PRMT5 inhibited the growth of prostate cancer cells. (**A**) The PRMT5 protein in LNCaP cells expressing PRMT5, PRMT5mt, NLS-PRMT5, or NLS-PRMT5mt was immunostained with the anti-PRMT5 antibody. The samples were observed under a confocal microscope. (**B**) Western blot of cytoplasmic (C) and nuclear (N) fractions of LNCaP cells expressing PRMT5, f:PRMT5mt, NLS-PRMT5, or NLS-PRMT5mt with anti-PRMT5, -p44, -lamin B, or -HSP90 antibody. (**C**) Growth curves of prostate cancer LNCaP cells expressing PRMT5, f:PRMT5mt, NLS-PRMT5, or NLS-PRMT5mt.

### RNA Interference

P44 shRNA (p44-shRNA) (target sequence: 5′-GGGAACTAGATGAGAATGA-3′), PRMT5 shRNA (target sequence: 5′-GGATAAAGCTGTATGCTGT-3′), and a nontargeting shRNA (NT-shRNA) (target sequence: 5′-TTCTCCGAACGTGTCACGT-3′) were designed with a hairpin and sticky ends (ClaI and MluI). The oligonucleotides were annealed into the lentiviral gene transfer vector, pLVTHM, using the ClaI and MluI restriction enzyme sites. The DNA constructs were sequenced to test for proper insertion and length of the inserts. The lentivirus was then produced by transfecting human embryonic kidney cells (293FT; Invitrogen) with the sequence-verified PLVTHM vector, the packaging plasmid (MD2G), and the envelope plasmid (PAX2), which are required for viral production. Three days later, the viral supernatant was collected and filtered to remove cellular debris. LNCaP cells (1×10^5^) were plated onto six-well plates and transduced with lentivirus vector particles. After 16 h, the virus-containing medium was removed and replaced with normal growth medium. Three days after infection, cells split at 1∶6 and were grown for 3 days. Whole-cell lysates (5 micrograms of protein) made from the infected cells were analyzed by Western blot.

**Figure 8:**
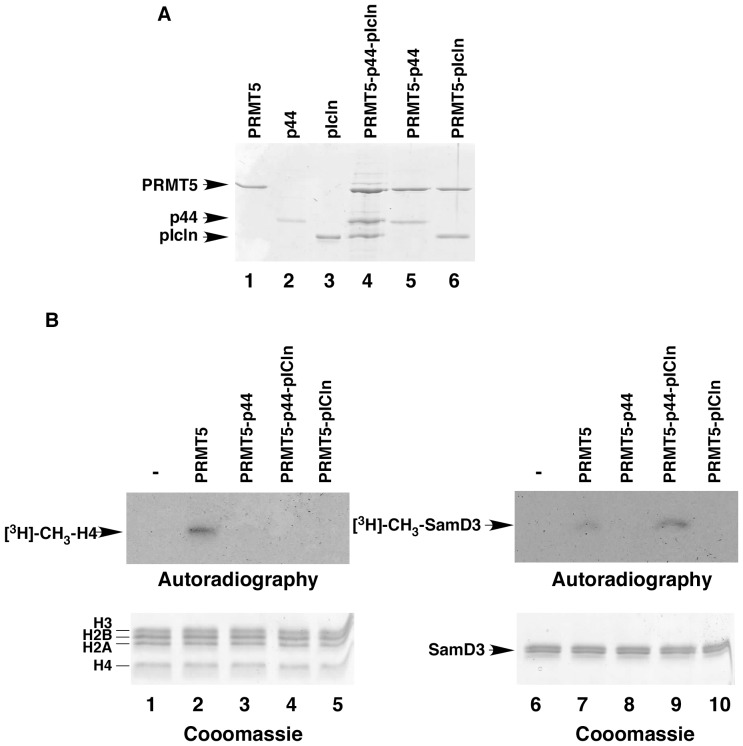
PRMT5 forms a stoichiometric complex with p44 and PRMT5. (**A**) SDS-PAGE of PRMT5 complexes produced by co-expression in *E. coli*. (**B**) Methylation of SmD3 and histone H4 substrates by PRMT5 and PRMT5-containing complexes. Top: autoradiography of the gel. Bottom: Coomassie blue staining of the gel.

### Nontargetable PRMT5 and p44 Expression

To create nontargetable PRMT5 and p44 expression vectors, the nucleotide sequences targeted by shRNAs were mutated using an oligo-direct mutagenesis kit. The target sequence GGATAAAGCTGTATGCTGT of PRMT5 shRNA was mutated to GGATAAAattaTATGCTGT. The target sequence GGGAACTAGATGAGAATGA of p44 was mutated to GGGAAtTgGAtGAGAATGA. The mutant PRMT5 or p44 cDNA was subcloned into the lentiviral expression vector (dsRed-OG2). The recombinant lentivirus was produced with 293T as described above. To rescue PRMT5 or p44 expression, LNCaP PRMT5-shRNA or p44-shRNA cells were plated onto six-well plates and transduced with the virus containing either the nontargetable PRMT5 or p44 expression vector or empty vector. After 48 h, the cells were re-plated, and the PRMT5 or p44 expression was confirmed by Western blot.

**Figure 9:**
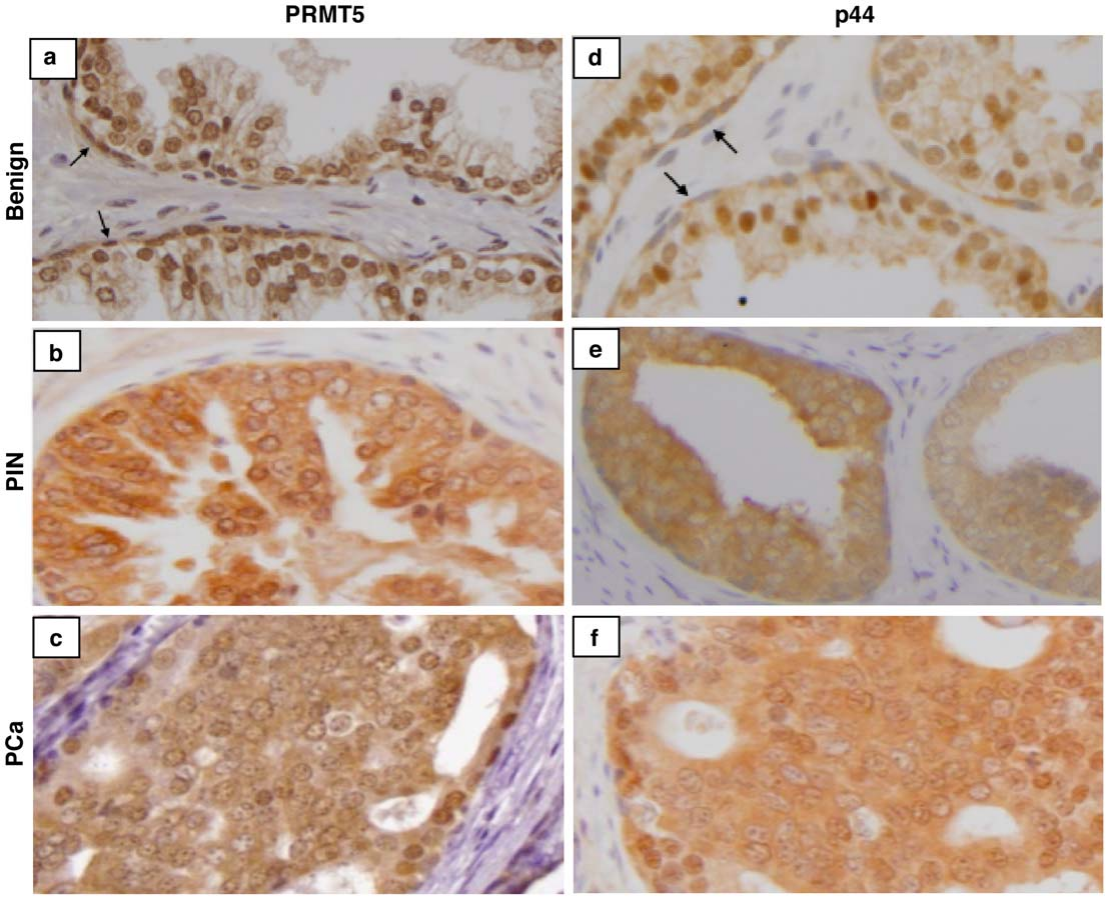
The distinct subcellular localization of PRMT5 and p44 in benign and malignant prostate tissues . Immunohistochemical staining of p44 and PRMT5 in human benign (top panels), prostatic epithelial hyperplasia (PIN, middle panels), and malignant prostate (PCa, Gleason grade 4, bottom panels) tissues.

### Cytoplasmic and Nuclear Extract Preparation

Cytoplasmic and nuclear fractions were prepared from cultured cells by using the Nuclear Extract Kit (catalogue #40010 and 40410, Active Motif) as described previously [28].

### Western Blot Analysis

PRMT5 and p44 were detected in total cell extracts (5 micrograms) by 10% SDS-PAGE and transferred to an Immobilon-P transfer membrane (Millipore). The membranes were washed in Tris-buffered saline with Tween 20 (10 mM Tris-HCl, pH 8, 150 mM NaCl, and 0.05% Tween 20) and blocked with 5% nonfat milk in Tris-buffered saline with Tween 20 for 1 h. The blots were then probed overnight with primary antibodies at dilutions of 1∶2,000 (anti-p44), 1∶1,000 (anti-PRMT5), 1∶1000 (anti-HSP90, Santa Cruz Biotechnology), 1;500 (anti-lamin B, Santa Cruz Biotechnology), and 1∶1,000 (anti-β-actin, Sigma-Aldrich). After 1.5 h of incubation with horseradish peroxide-conjugated secondary antibody, immunoreactive proteins were detected by enhanced chemiluminescence using the ECL detection system per the manufacturer's instructions (GE Healthcare). Protein concentrations were determined by using the Bradford protein assay (Bio-Rad).

### Co-immunoprecipitation

PC3 cells (3.6×10^6^) were transfected with 6 micrograms of pcDNA-f:p44, -f:p44(26–27AAA), -f:p44(29–31AAA), -f:p44(35–37AAA), or -f:p44(42–44AAA) with Lipofectamine 2000. The whole-cell lysates were prepared from the transfected cells 48 h after transfection and incubated with 15 microliters of M2 agarose (Sigma) for 2 h at 4°C in a final volume of 0.5 ml containing 20 mM HEPES (pH 7.9), 0.2 mM EDTA, 20% glycerol, 2 mM DTT, 300 mM KCl, and 0.1% NP40. The beads were washed five times (1 ml each) with the incubation buffer. The bound proteins were eluted with 30 microliters of FLAG peptides (0.2 mg/ml) for 30 min at 4°C and analyzed by Western blot with anti-PRMT5 antibody.

### Protein Expression and Purification

PRMT4, p44, pICln, or SmD3 cDNA was cloned into pET15d (Novagen) to be expressed as an amino-terminal His_6_-tagged protein. For PRMT5 co-expression with p44 or pICln, PRMT5 was cloned into pACYCDuet (Novagen) with an amino-terminal His_6_ tag, and the p44 or pICln coding region was cloned into the second multiple cloning site of the same vector with a N-terminal FLAG-epitope tag. For PRMT5 co-expression with p44 and pICln, PRMT5 was cloned into pACYCDuet with an amino-terminal His_6_ tag, the pICln coding region was cloned into the second multiple cloning site of the same vector, and the coding region of p44 was cloned into a pET vector with an N-terminal FLAG-epitope tag. Proteins were expressed in BL21(DE3) cells at 30°C for 3 h after induction with 0.1 mM isopropyl 1-thio-β-D-galactopyranoside. Cells were lysed by sonication three times for 5 min each in lysis buffer (10 mM HEPES, pH 7.9, 0.3 M KCl, 0.1% NP40, 0.1 mM phenylmethylsulfonyl fluoride, 2 βg/ml pepstatin A, and 2 micrograms/ml leupeptin). The proteins were purified using Ni-NTA agarose (Qiagen) according to the manufacturer's protocol with imidazole elution and subsequently purified on the M2 agarose (Sigma-Aldrich) with FLAG peptide elution for PRMT5-containing complexes. Histones were purified from HeLa cells as described previously [24].

### Methyltransferase Assay

Methylation reactions were performed as previously described with a few modifications [24]. Reactions containing 6 fmol of PRMT5 or PRMT5-containing complexes, 1 microgram of SmD3 or histones, and 1 microCi of S-[methyl-^3^H]adenosymethionine (PerkinElmer) were incubated in 50 mM Tris-HCl, pH 7.5, 1 mM EGTA, and 1 mM EDTA at 30°C for 1 h. Reactions were boiled in SDS sample buffer and separated on a 15% polyacrylamide gel. Gels were fixed for 30 min in 40% methanol-10% acetic acid, incubated in 20 ml of Amplify (Amersham Life Science) for 10 min, dried, and exposed to x-ray film at −80°C.

## Results

### PRMT5 and p44 Co-localized in the Cytoplasm of Prostate Cancer Cells

The subcellular localization of PRMT5 is dynamically regulated during mouse development [8]. It localizes to the nucleus during early development and is found in the cytoplasm of the pluripotent epiblast cells of the inner cell mass by embryonic day 6.5. PRMT5 primarily localizes to the cytoplasm in somatic cells such as 293T, Cos-1, U2OS, and normal B-cells [35], [38], [39]. In our current study, immunostaining with the anti-PRMT5 antibody indicated that PRMT5 is predominantly cytoplasmic in prostate cancer PC3 and LNCaP cells (Fig. 1A, panels a and d).

Western blot of the cytoplasmic and nuclear fractions confirmed its subcellular localization (Fig. 1B, top panel). PRMT5 was also detected in the nuclei of PC3 and LNCaP cells as separate nuclear bodies (Fig. 1A, panels c, f, and g, indicated by white arrows).

The nucleus contains many dynamic nuclear structures, including Cajal bodies [40]. Although the function of the Cajal body remains unknown, there is considerable evidence suggesting that it may be involved in snRNA maturation/biogenesis, histone pre-mRNA processing, and the assembly of the transcriptosomes [41], [42]. The Cajal body contains many components including coilin (the marker of Cajal bodies), snRNPs, and SMN. PRMT5, MEP50, pICln, and Sm proteins form the methylosome complex that mediates the assembly of spliceosomal snRNP [18], [20]. SMN-complex, containing the Sm proteins and PRMT5, is necessary and sufficient for assembly of UsnRNA [21], [43]. We immunostained LNCaP cells using a rabbit anti-coilin and a mouse anti-PRMT5 antibody. The merged image demonstrated that PRMT5 is not co-localized with Cajal bodies in the nucleus (Fig. 1A, panel h).

In agreement with our previously reported data [25], p44 protein localized predominantly in the cytoplasm of prostate cancer PC3 and LNCaP cells (Fig. 1A, panels b and e; Fig. 1B, 2nd panel). The merged images demonstrated a good co-localization of PRMT5 with p44 in the cytoplasm, whereas this co-localization was not observed in the nucleus of PC3 and LNCaP cells (Fig. 1A, panels c, f, and g).

### PRMT5 Contains Three Nuclear Exclusion Signals

The N-terminal enhanced green fluorescent protein (GFP)-tagged PRMT5 (GFP-PRMT5) was transiently expressed in PC3, LNCaP, and Cos 7 cells, and the resulting GFP-PRMT5 fusion protein had a predominant cytoplasmic localization (Fig. 2B, panels a and e; data not shown for PC3 cells) in those cells, similar to that of the endogenous PRMT5 protein (Fig. 1A, panel d). The GFP protein localizes in both cytoplasm and nucleus in these cells (Fig. 2B, panels I and j). To identify the molecular determinant for subcellular localization of PRMT5, overlapping fragments spanning the entire open-reading frame of PRMT5 (Fig. 2A) were cloned in frame to generate pcDNA-f:GFP-PRMT5 fusion constructs. These constructs were transfected into Cos 7 cells to determine the critical regions of PRMT5 necessary for nuclear export or import.

Two protein fragments, PRMT5(1–324) and PRMT5(325–637), were found within the cytoplasm in 100% of transfected cells (Fig. 2A), suggesting that these fragments contain signals required for cytoplasmic localization. Deletion of 144 or 234 amino acid residues from the C-terminal end of the PRMT5(1–324) fragment did not affect its cytoplasmic localization. Further deletions of six or seven amino acid residues from the N-terminal or C-terminal led to complete loss of cytoplasmic localization, indicating that these amino acid residues are critical for cytoplasmic localization of this fragment. The region PRMT5(1–90) was found within the cytoplasm in 100% of transfected cells (Fig. 2A; 2B, panel b) and is a novel NES, designated NES1. NES1 does not resemble the conventional leucine-rich NES [44].

Further deletion analysis identified the other two NES sequences in the C-terminal part of PRMT5. The region spanning amino acid residues 500 to 560 localized to the cytoplasm in 100% of transfected cells (Fig. 2A; 2B, panel c). Thus, the PRMT5(500–560) fragment is also a functional NES, designated NES2. The region spanning amino acid residues 576 to 637 localized to the cytoplasm in 98% of transfected cells (Fig. 2A; 2B, panel d) and is another functional NES, designated NES3. Sequence analysis indicated that these two NES sequences are novel and also do not resemble the classical leucine-rich NES [44]. Western blot analysis of cytoplasmic and nuclear fractions of transfected cells confirmed the cytoplasmic localization of the full-length PRMT5 proteins and identified NESs (Fig. 3C, top panel). No nuclear localization signals (NLSs) were detected in the PRMT5 protein by this analysis. The identified NESs functioned similarly in LNCaP cells (Fig. 2B, middle panels) and PC3 cells (data not shown).

### PRMT5 Promotes Cytoplasmic Translocation of p44

PRMT5 physically interacts and forms a complex with p44/MEP40/WD45/WDR77 in various cells, including prostate cancer cells [33], [34], [35], and co-localized with p44 in the cytoplasm of prostate cancer cells (Fig. 1A). We then investigated whether PRMT5 expression influences subcellular localization of p44. Cos 7 cells were transfected with pcDNA-GFP-p44 alone or together with pcDNA-PRMT5. Consistent with previous published results [28], strong GFP-p44 signals were evident in the nucleus in transfected Cos 7 cells (Fig. 3B, panel a). However, co-expression of PRMT5 resulted in exclusive cytoplasmic localization of GFP-p44 in 100% of transfected cells (Fig. 3A; 3B, panel b). Deletion analysis indicated that the C-terminal part (amino acid residues 325–637) is essential and sufficient to promote GFP-p44 cytoplasmic translocation (Fig. 3A; 3B, panel d). The PRMT5-driven cytoplasmic translocation of p44 was confirmed by Western blot analysis of the cytoplasmic and nuclear fractions of transfected cells (Fig. 3C, top panel). Similar observations were obtained with LNCaP and PC3 cells (Fig. 3B, bottom two panels). The conserved arginine residue (R368) is essential for the methyltransferase activity of PRMT5 [45]. The mutation of R368A on PRMT5 abolished its methyltransferase activity [24] but did not affect its ability to promote p44 cytoplasmic translocation (Fig. S1). Thus, PRMT5 is the primary force in determining the cytoplasmic localization of the PRMT5-p44 protein complex.

### The PRMT5-p44 Interaction is Required for the PRMT5-driven Cytoplasmic Translocation of p44

Deletion analysis indicated that amino acid residues 26 to 45 in the p44 protein were critical for the PRMT5-driven cytoplasmic translocation of p44 (Fig. S2). We mutated the amino acid residues (^26^CME^28^, ^29^RQL^31^, ^35^RYR^37^, or ^42^LLL^44^) to alanines in the p44 protein and examined the consequence of these mutations on PRMT5-driven cytoplasmic translocation of GFP-p44 (Fig. 4A). These mutations did not change the GFP-p44 subcellular localization in Cos 7 cells (Fig. 4A, panels g, m, s, y versus a). However, mutations (^35^RYR^37^ to ^35^AAA^37^ and ^42^LLL^44^ to ^42^AAA^44^) abolished PRMT5-driven cytoplasmic translocation of GFP-p44 (Fig. 4A, panels v, b' versus d).

These mutations were expressed as FLAG epitope-tagged proteins in PC3 cells. The mutations decreased expression levels of the p44 protein (Fig. 4B, top panel, lanes 2–5 versus lane 1). Immunoprecipitation with the anti-FLAG antibody immobilized on agarose beads (M2-agarose) indicated that the mutations (^35^RYR^37^ to ^35^AAA^37^ and ^42^LLL^45^ to ^42^AAA^44^) in p44 abolished its interaction with PRMT5 (Fig. 4B, bottom panel, lanes 4 and 5 versus lanes 1–3). Taken together, these results suggest that the interaction between p44 and PRMT5 is essential for the PRMT5-driven cytoplasmic localization of p44.

### Both PRMT5 and p44 are Required for Growth of Prostate Cancer Cells

To determine whether PRMT5 plays a role in prostate cancer, we tested whether silencing PRMT5 expression in LNCaP cells would affect their growth. To do so, we designed a short hairpin-interfering RNA (shRNA) targeted against the PRMT5 sequence. To test whether the shRNA could suppress PRMT5 expression, we infected LNCaP cells with the lentiviral vector transducing a DNA segment specifying such shRNA sequence. As shown in Fig. 5A, the shRNA dramatically reduced expression of PRMT5 protein in LNCaP cells 4 days after the lentivirus infection (lane 2, top panel) compared with that expression by a non-target (NT) shRNA (lane 1, top panel), whose sequence did not match any known human gene. Similarly, p44 shRNA also dramatically decreased p44 protein levels in LNCaP cells (Fig. 5A, lane 6 versus lane 5, top panel).

LNCaP cells expressing NT, PRMT5, or p44 shRNA were plated onto 24-well plates, and cell numbers were counted every day. Silencing PRMT5 or p44 expression strongly inhibited the growth of LNCaP cells (Fig. 5B). LNCaP cells were first infected with lentivirus expressing PRMT5 or p44 shRNA and 2 days later infected with lentivirus expressing the shRNA-resistant PRMT5 or p44. The infected cells were grown for 4 days and submitted for Western blot (Fig. 5A, lane 3) and cell growth assay (Fig. 5B). Expression of the shRNA-resistant PRMT5 or p44 completely restored the growth of LNCaP cells expressing PRMT5 or p44 shRNA. Thus, both PRMT5 and p44 are required for the growth of prostate cancer cells. The shRNA-resistant mutant (R368A) PRMT5 failed to restore growth inhibition induced by PRMT5 shRNA (Fig. 5B), indicating that the methyltransferase activity of PRMT5 is required for the growth of LNCaP cells. Western blot analysis with anti-PRMT5 antibody showed that the mutant PRMT5 was expressed at higher levels than the wild-type PRMT5 in LNCaP cells (Fig. 5A, lane 4 versus lanes 3 and 1, top panel).

### PRMT5 and p44 are Co-expressed in the Cytoplasm

We noticed that the mutant p44 proteins that lack the interaction with PRMT5 were expressed at lower levels than the wild-type p44 protein (Fig. 4B), and silencing PRMT5 expression also downregulated p44 expression in LNCaP cells (Fig. 5A, lane 2, second panel), which could be restored by wild-type or methyltransferase activity-deficient PRMT5 expression (Fig. 5A, lanes 3 and 4, second panel). Thus, p44 expression may be dependent on PRMT5. To investigate this possibility, LNCaP cells expressing NT shRNA or PRMT5 shRNA were submitted for immunostaining for PRMT5 (Fig. 6A, panels a-f) or p44 (Fig. 6A, panels g-l). As expected, PRMT5 signals were significantly lower in the PRMT5 shRNA-expressing cells (Fig. 6A, 2nd panels versus top panels). The p44 protein levels were also significantly decreased in the PRMT5 shRNA-expressing cells (4th panels versus 3rd panels). Western blot analysis of the cytoplasmic and nuclear fractions of LNCaP cells expressing NT shRNA or PRMT5 shRNA indicated a significant decrease in PRMT5 expression in the cytoplasm of PRMT5 shRNA-expressing cells (Fig. 6B, top panel, lane 3 versus lane 1). PRMT5 silencing also dramatically decreased p44 expression in the cytoplasm (Fig. 6B, bottom panel, lane 3 versus lane 1) but slightly increased the p44 protein in the nuclear fraction (Fig. 6B, bottom panel, lane 4 versus lane 2). On the other hand, silencing p44 led to a dramatic decrease in p44 protein levels in the cytoplasm but barely affected the nuclear p44 expression (top panel, lane 7 versus lane 1, lane 8 versus lane 6). Silencing p44 also resulted in a significant decrease in cytoplasmic PRMT5 levels (bottom panel, lane 7 versus lane 1) and had little effect on the nuclear PRMT5 (bottom panel, lane 8 versus lane 6). These results suggest that PRMT5 and p44 are co-expressed in the cytoplasm of prostate cancer cells.

PRMT5 or p44 was silenced in LNCaP cells, and at the same time the shRNA-resistant p44 or PRMT5 was expressed via lentivirus. As demonstrated above, p44 or PRMT5 shRNA expression strongly downregulated both PRMT5 and p44 protein levels (Fig. S3A, lanes 2 and 3) and inhibited the growth of LNCaP cells (Fig. S3B). The lentivirus-mediated expression of PRMT5 increased the PRMT5 protein to a level comparable to that in control cells (Fig. S3A, lane 4 versus lane 1) but did not alter the p44 protein level in p44 shRNA-expressing cells (lane 4 versus lane 2). However, PRMT5 expression along with low levels of the p44 protein did not restore growth inhibition induced by silencing p44 expression in LNCaP cells (Fig. S3B). We failed to express p44 in the PRMT5 shRNA-expressing LNCaP cells (Fig. S3A, lane 5) and could not determine whether p44 expression with low levels of the PRMT5 proteins can restore growth inhibition induced by silencing PRMT5 in LNCaP cells. These results suggest that PRMT5 and p44 not only physically co-exist but also function together in the cell cytoplasm.

### The Nuclear PRMT5 Inhibited Growth of Prostate Cancer Cells

To investigate the function of PRMT5 in the nucleus, we targeted PRMT5 into the nucleus of LNCaP cells by fusing a strong nuclear localization signal (NLS) [25] at the N-terminal end of PRMT5. LNCaP cells were infected with lentivirus expressing PRMT5, PRMT5mt (R386A), NLS-PRMT5, or NLS-PRMT5mt. The exogenous PRMT5 and PRMT5mt proteins mainly localized in the cytoplasm of LNCaP cells (Fig. 7A, panels b and c; Fig. 7B, lanes 3–6, top panel). NLS-PRMT5 and NLS-PRMT5mt expression dramatically increased the number and density of bright foci in the nucleus of LNCaP cells (Fig. 7A, panels d and e) as well as PRMT5 protein levels in the nuclear fraction (Fig. 7B, lanes 7–8; data not shown for PRMT5mt). The doubling time of the control lentivirus-transfected LNCaP cells (control) was about 30 h, which was similar to that of the parental LNCaP cells (Fig. 7C). The doubling times for PRMT5- and PRMT5mt-expressing cells were similar to that for the control cells. However, the doubling time for NLS-PRMT5- and NLS-PRMT5mt-expressing cells was about 72 h. These results indicated that the nuclear PRMT5 (NLS-PRMT5 and NLS-PRMNT5mt) inhibited the growth of LNCaP cells in a methyltransferase activity-independent manner. PRMT5 overexpression increased PRMT5 protein levels in the cytoplasm (Figure 7C, lane 3 versus lane 1) as well as in the nucleus (Figure 7C, lane 4 versus lane 2). Given that fact that PRMT5 has opposite effects on cell growth when localized in the cytoplasm and nucleus, the effect of PRMT5 overexpression on LNCaP cell growth was neutral.

### PRMT5 Forms a Complex with p44 and pICln with Distinct Substrate Specificity

Several groups have shown that PRMT5 interacts with p44 and pICln [33], [34], [35] and that PRMT5 forms a stoichiometric complex with pICln and Sm proteins [46], but whether PRMT5, p44, and pICln form a stoichiometric complex has not been reported. To test whether PRMT5 can form complexes with p44 or/and pICln, we co-expressed and co-purified PRMT5 with p44 and/or pICln. We found that PRMT5 can be co-expressed and co-purified with p44 or pICln (Fig. 8A, lanes 5 and 6). By employing these co-expression approaches, we were able to produce a stoichiometric PRMT5-p44-pICln complex (Fig. 8A, lane 4).

To test the methyltransferase activity, we incubated purified PRMT5 or PRMT5-containing complexes with SmD3 or histone substrate in the presence of S-[methyl-^3^H]adenosymethionine. SmD3 contains multiple RG repeats in its carboxyl terminus that are methylated by PRMT5 *in vivo* and *in vitro* [16], [46], [47]. PRMT5 methylates the third arginine residue in histone H4 [1]. PRMT5 alone can methylate SmD3 as well as histone H4 (Fig. 8B, lanes 2 and 7). However, PRMT5-p44 and PRMT5-pICln complexes did not show any detectable level of methyltransferase activity (Fig. 8B, lanes 3, 5, 8, and 10), suggesting that p44 or pICln inhibits PRMT5 when either is pre-bound to the enzyme. Previous study also demonstrated that pICln alone inhibited PRMT5 methyltransferase activity [46]. Interestingly, the PRMT5-p44-pICln complex showed PRMT5 methyltransferase activity with SmD3 (Fig. 8B, lane 9) but not with histone H4 substrate (Fig. 8B, lane 4).

### Distinct PRMT5 and p44 subcellular localization in benign prostate and prostate cancer

To evaluate p44 and PRMT5 nuclear and cytoplasmic subcellular localization in areas of benign, premalignant (prostatic intraepithelial neoplasia [PIN]), and cancer tissues, we performed immunohistochemical staining for p44 and PRMT5 in 19 samples derived from patients with prostate cancer. Both p44 and PRMT5 were negative in stromal cells (Fig. 9). As observed previously [25], p44 was expressed in the nucleus of benign prostate epithelial cells (Fig. 9, panel d) and in the cytoplasm of cells in premalignant prostate lesions, PIN (panel e), and cancer (panel f). Similarly, PRMT5 was localized in the nucleus of benign prostate epithelial cells (panel a) and in the cytoplasm of cells in high-grade PIN (panel b) and cancer (panel c). The patterns of p44 and PRMT5 expression in benign prostate epithelial cells and in PIN and prostate cancer cells were almost identical in terms of subcellular localization, consistent with our previous report of p44 and PRMT5 co-localization in the testis [36]. However, PRMT5 was also expressed in the nucleus of the basal cells (panel a, indicated by black arrows) but p44 was only weakly detected in the cytoplasm of the basal cells (panel d, indicated by black arrows), indicating that PRMT5 has p44-independent functions in basal cells. These staining patterns were observed with all 19 samples utilized.

## Discussion

By using a subcellular localization assay, we found that PRMT5 contains three strong NES sequences that determine its predominant cytoplasmic localization in prostate cancer cells. The functional activity of PRMT5 is controlled by its subcellular localization. When localized in the cytoplasm of prostate cancer cells, PRMT5 is essential for cell growth; in contrast, the nuclear PRMT5 suppresses cell growth. PRMT5 and p44 are co-expressed in the cytoplasm, and both are required for the growth of prostate cancer cells. In addition, we demonstrated that PRMT5 and p44 localized in the nucleus in benign prostate epithelium but localized in the cytoplasm in prostate premalignant and cancer tissues. These results imply a novel role for PRMT5 and p44 in the control of cell growth and in prostate tumorigenesis.

### PRMT5 Has Three Nuclear Exclusion Signals

Mammalian PRMT5 primarily localizes to the cytoplasm in somatic cells [35], [38], [39] and in cord blood progenitors and mouse primordial germ cells after embryonic day 11.5 [12], [48]. PRMT5 is highly expressed in both the nucleus and cytoplasm in transformed mantle cell lymphomas and enriched in the nucleus in patient samples [39]. It has been proposed that PRMT5 relocates from the nucleus to the cytoplasm, where it may play a role in regulating pluripotency [49], [50]. However, little is known of the signals that control PRMT5 subcellular translocation. By studying various PRMT5 segments that determine nuclear or cytoplasmic localization of fused GFP, we defined three novel NES sequences in the PRMT5 protein. These NESs are the basis for the cytoplasmic localization of PRMT5. No nuclear localization activity was detected in the full-length PRMT5 or various PRMT5 truncations. These results are consistent with the fact that PRMT5 translocation from the cytoplasm to the nucleus is dependent on co-expression of AJUBA and SNAIL in U2OS cells [35], and its relocation from the nucleus to the cytoplasm may be due to the loss of its binding partner Blimp1 in the case of differentiation of mouse primordial germ cells and human fetal gonocytes [12], [50], [51].

The p44 protein localizes to both the cytoplasm and nucleus of prostate cancer cells when expressed as a GFP-fusion protein since it contains both NESs and NLSs [28]. However, co-expression of PRMT5 resulted in exclusive p44 cytoplasmic localization. PRMT5-promoted p44 cytoplasmic translocation is dependent on the interaction between PRMT5 and p44 and is not dependent on the methyltransferase activity of PRMT5. Understanding the exact mechanism of how these two proteins co-localize in the cytoplasm will require further investigation. One possibility is that PRMT5 somehow blocks the nuclear import functions of p44 by masking or sequestering its NLSs.

### The Subcellular Localization of PRMT5 Affects Its Function

Silencing PRMT5 or p44 by shRNA significantly decreased both PRMT5 and p44 proteins in the cytoplasm and had little effect on their expression in the nucleus of LNCaP cells. The slow turnover of proteins in the nucleus might account for this different effect of shRNAs on cytoplasmic and nuclear p44 and PRMT5 proteins. Silencing p44 or PRMT5 expression dramatically inhibited the growth of LNCaP cells. The growth effect of PRMT5 is dependent on its methyltransferase activity. Given the fact that p44 and PRMT5 form a stoichiometric complex and are co-expressed in the cytoplasm of LNCaP cells, it is more likely that p44 and PRMT5 (probably also including pICln) in the cytoplasm function as a unit (complex) in the control of cell growth via methylating substrates.

On the other hand, the forced nuclear localization of p44 [25], [26] or PRMT5 inhibited the growth of prostate cancer cells. The growth inhibition mediated by PRMT5 is independent of its methyltransferase activity. Because PRMT5 and p44 are not co-localized in the nucleus, the mechanisms by which they suppress cell growth might be different. PRMT5 alone can methylate histone H4 and p44 suppressed this activity, further suggesting that PRMT5 functions in the nucleus in a p44-independent manner. A nuclear protein, called cooperator of PRMT5 (COPR5), tightly bound to PRMT5 both *in vitro* and in living cells [52]. PRMT5 bound to COPR5 methylates histone H4 (R3), and COPR5 depletion in cells strongly reduced PRMT5 recruitment on chromatin at the PRMT5 target gene cyclin E1 (CCNE1) *in vivo*. Thus, COPR5 may be an important chromatin adaptor for PRMT5 to function in the nucleus.

Our studies revealed distinct subcellular localization of p44 [25], [26] and PRMT5 during prostate tumorigenesis. In the human prostate, PRMT5 and p44 are resident in the nucleus of benign epithelial cells, whereas in prostate cancer cells, PRMT5 and p44 localize in the cytoplasm. This translocation event occurs in hyperplastic epithelial cells in PIN. Because cytoplasmic PRMT5 and p44 are required for the growth of prostate cancer cells, and nuclear PRMT5 and p44 inhibit cancer cell growth, nucleocytoplasmic transport of PRMT5 and p44 might be essential event during prostate tumorigenesis.

Taken together, the results from this study provide insight into the functional roles of PRMT5 in the control of cell growth and in prostate tumorigenesis.

## Supporting Information

Figure S1 **PRMT5 promotes p44 cytoplasmic translocation independent of its methyltransferase activity.** (**A**) Cells were transfected with pcDNA-GFP-p44 and pcDNA-PRMT5 or pcDNA-PRMT5mt and the GFP-p44 subcellular localization was observed under a confocal microscope. (**B**) Western blot analysis of PRMT5 and PRMT5mt expression in the transfected cells.
(TIF)

Figure S2 **The amino acid residues in p44 are required for PRMT5-promoted p44 cytoplasmic translocation.** Cells were transfected with pcDNA-PRMT5 and pcDNA-GFP-p44 or pcDNA-GFP-p44 truncations, and the GFP-p44 or GFP-p44 truncation subcellular localization was observed under a confocal microscope.
(TIF)

Figure S3 **PRMT5 expression alone is not sufficient to support growth of LNCaP cells.** (**A**) Western blot of whole-cell lysates derived from LNCaP cells expressing NT shRNA, p44 shRNA, PRMT5 shRNA, p44 shRNA plus PRMT5, PRMT5 shRNA plus p44 with anti-PRMT5, -p44, or -actin antibody as indicated. (**B**) Growth curves of LNCaP cells expressing NT shRNA, p44 shRNA, PRMT5 shRNAs, p44 shRNA plus PRMT5, or PRMT5 shRNA plus p44.
(TIF)
