# Supplementary figures and images for: Protein Arginine Methyltransferase 5 Functions in Opposite Ways in the Cytoplasm and Nucleus of Prostate Cancer Cells
Source: PLoS One. 2012 Aug 27;7(8):e44033. doi: 10.1371/journal.pone.0044033 (PMC3428323; doi:10.1371/journal.pone.0044033)

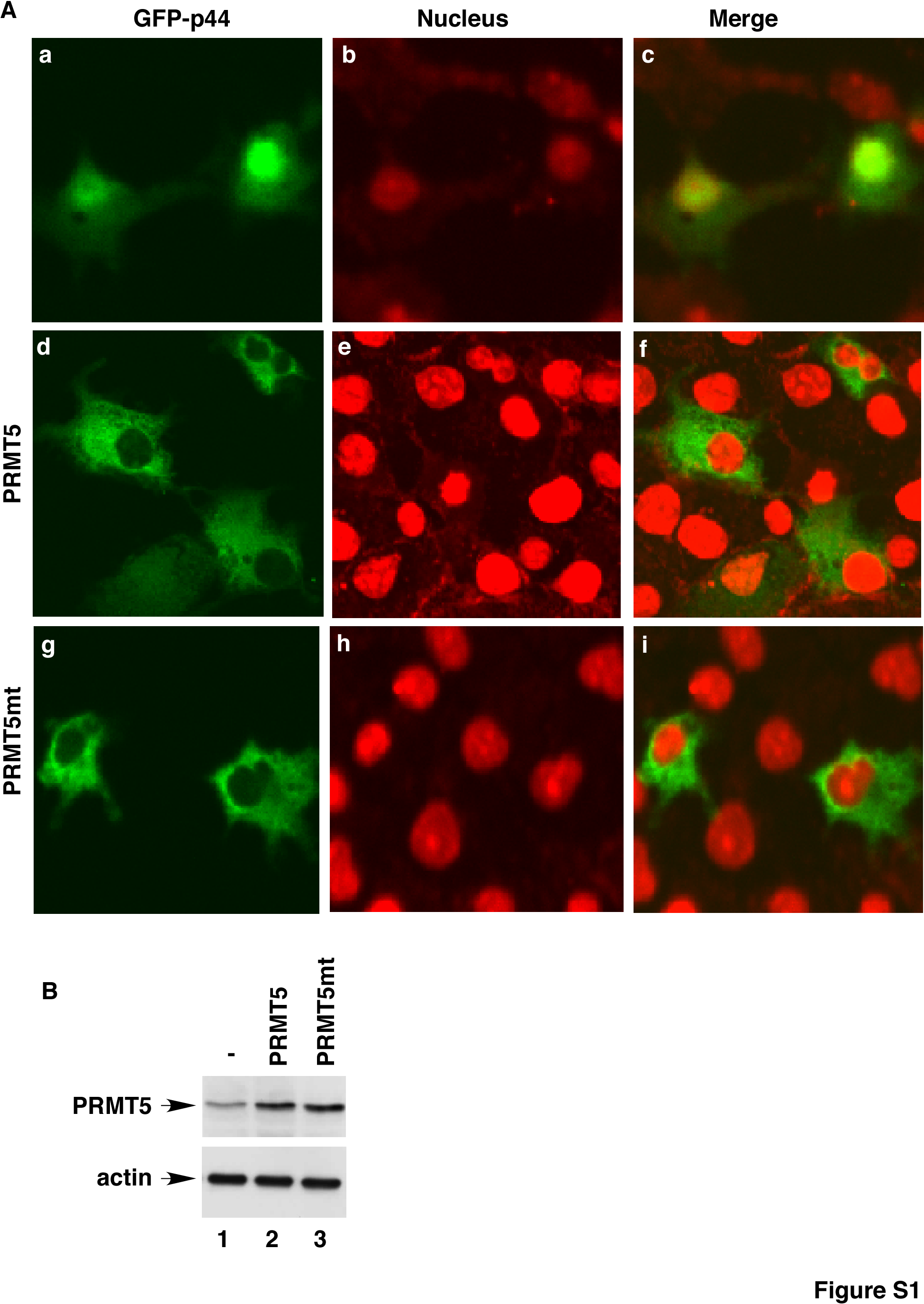

Supplement: Figure S1 — PRMT5 promotes p44 cytoplasmic translocation independent of its methyltransferase activity. (A) Cells were transfected with pcDNA-GFP-p44 and pcDNA-PRMT5 or pcDNA-PRMT5mt and the GFP-p44 subcellular localization was observed under a confocal microscope. (B) Western blot analysis of PRMT5 and PRMT5mt expression in the transfected cells. (TIF) [file pone.0044033.s001.tif]

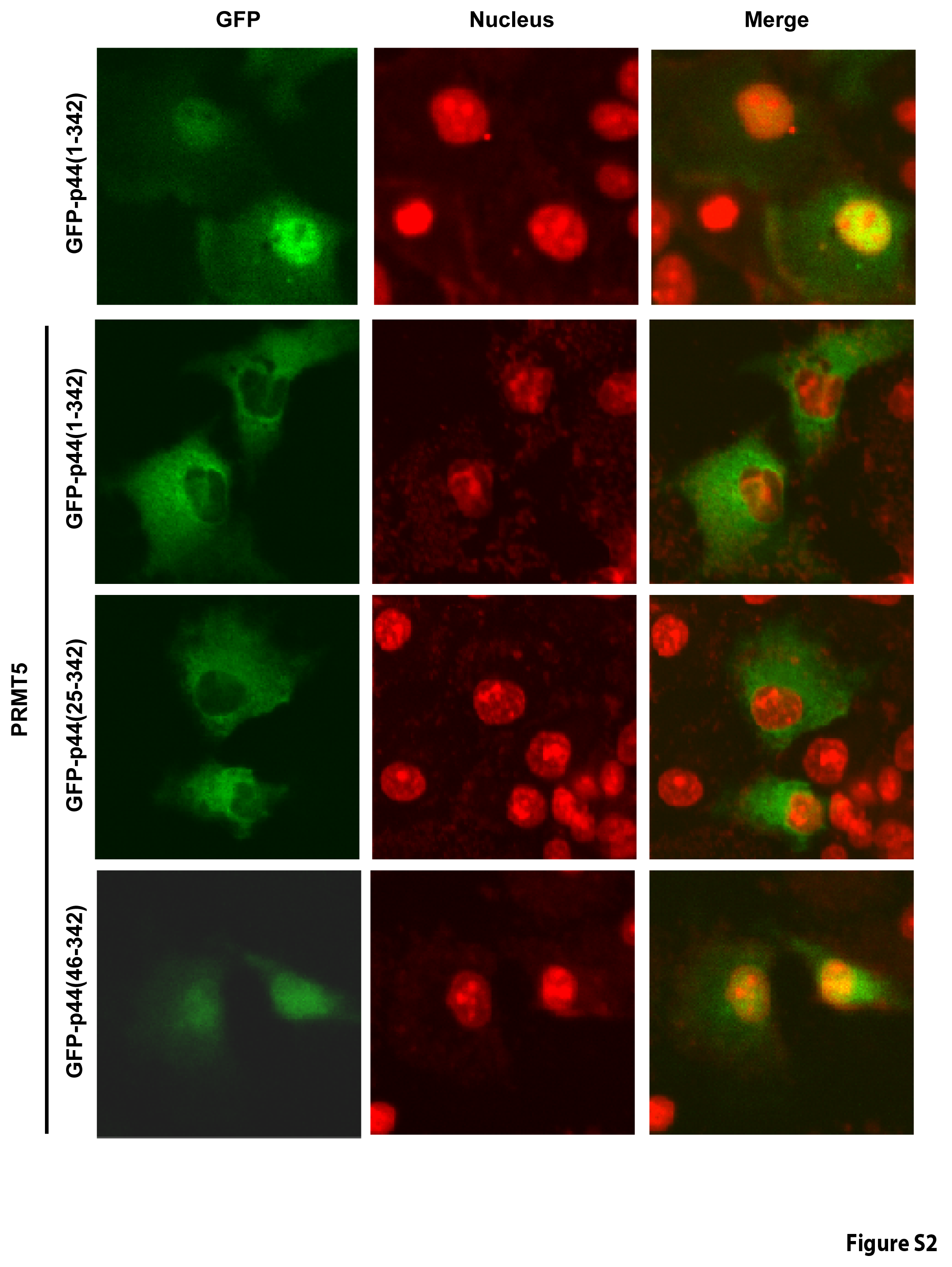

Supplement: Figure S2 — The amino acid residues in p44 are required for PRMT5-promoted p44 cytoplasmic translocation. Cells were transfected with pcDNA-PRMT5 and pcDNA-GFP-p44 or pcDNA-GFP-p44 truncations, and the GFP-p44 or GFP-p44 truncation subcellular localization was observed under a confocal microscope. (TIF) [file pone.0044033.s002.tif]

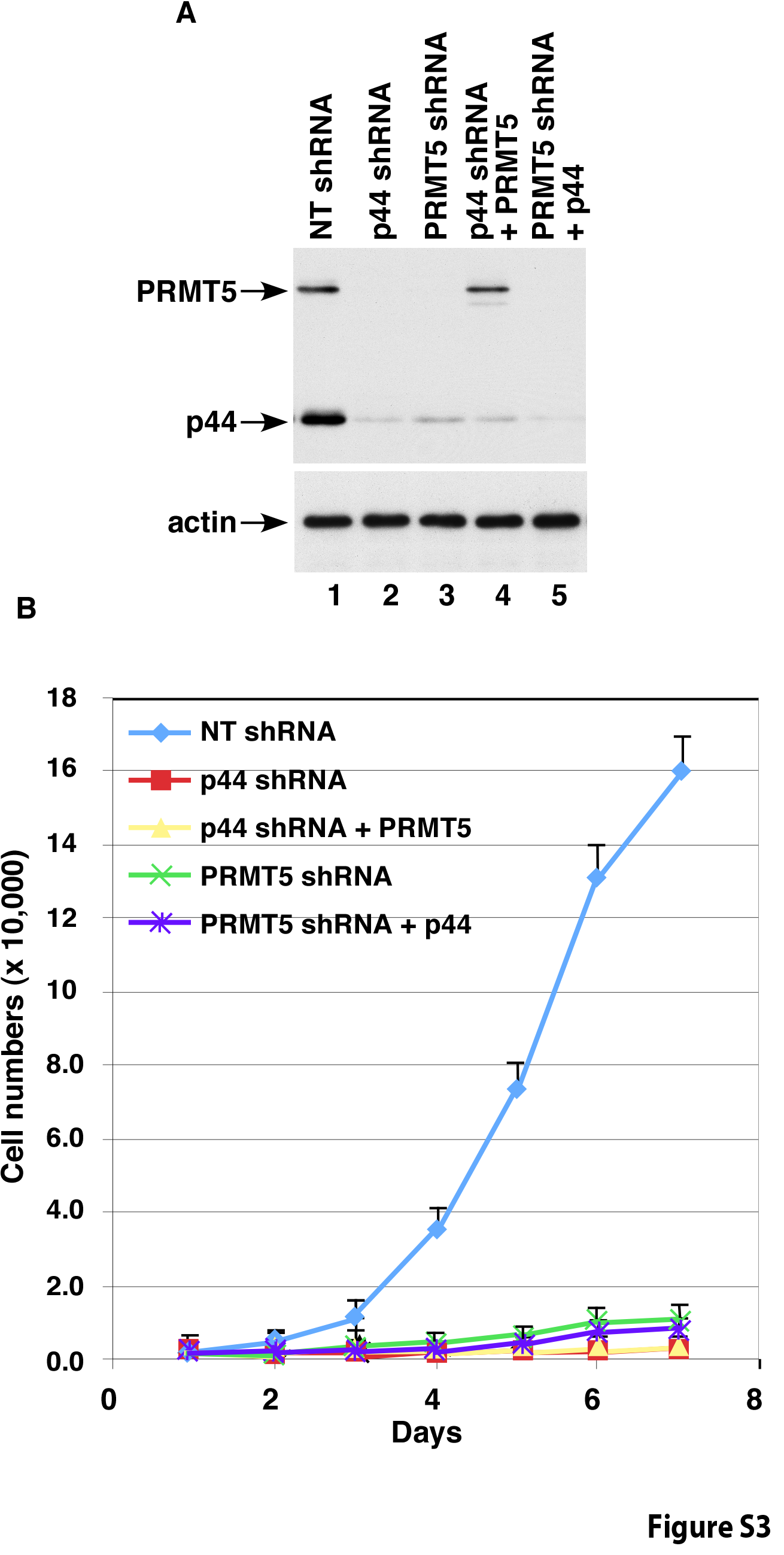

Supplement: Figure S3 — PRMT5 expression alone is not sufficient to support growth of LNCaP cells. (A) Western blot of whole-cell lysates derived from LNCaP cells expressing NT shRNA, p44 shRNA, PRMT5 shRNA, p44 shRNA plus PRMT5, PRMT5 shRNA plus p44 with anti-PRMT5, -p44, or -actin antibody as indicated. (B) Growth curves of LNCaP cells expressing NT shRNA, p44 shRNA, PRMT5 shRNAs, p44 shRNA plus PRMT5, or PRMT5 shRNA plus p44. (TIF) [file pone.0044033.s003.tif]
